# Biotin-switch technique/proximity ligation assay-based method for *in situ* analysis of S-nitrosylated proteins

**DOI:** 10.1016/j.redox.2026.104251

**Published:** 2026-06-08

**Authors:** Mélina Meunier, Emma Levieux, Stéphanie Plenchette

**Affiliations:** a Université Bourgogne Europe, EPHE, INSERM, CTM UMR 1231, 21000 Dijon, France; b EPHE, PSL University, Paris, France

**Keywords:** Nitric oxide, S-nitrosylation, Biotin-switch technique, Proximity ligation assay, Signaling

## Abstract

Nitric oxide (NO) influences a multitude of physiological and pathological processes. A major part of NO signaling involves S-nitrosylation (SNO), a post-translational modification in which an NO moiety is added to a selective cysteine thiol of a protein to form an S-nitrosothiol. *Per se*, S-nitrosylation is implicated in the control of tissue homeostasis and dysregulated basal levels of S-nitrosylation can contribute to malignant diseases. Importantly, exploiting NO bioactivity through S-nitrosylation represents a therapeutic interest in many diseases. S-nitrosylated protein analysis has been the subject of various methods of detection, quantification and identification, and the Biotin-Switch Technique (BST) is the reference method. Here, a new methodology combining BST and Proximity Ligation Assay (PLA) is presented for the *in situ* analysis of selective protein S-nitrosylation. This newly developed method termed “SNO-Biotin-PLA” provides the advantage of filling a gap in current methods for studying protein S-nitrosylation *in situ*.

## Highlights

• SNO-Biotin-PLA is a new methodology for the *in situ* analysis of selective protein S-nitrosylation.
• Low cell input is sufficient for *in situ* analysis of S-nitrosylation.
• SNO-Biotin-PLA enables discrimination between extracellular and intracellular S-nitrosylation in transmembrane proteins.

## Abbreviations:

**BST**: Biotin-Switch Technique
**DNA**: DeoxyriboNucleic Acid
**EDTA**: Ethylenediaminetetraacetic acid
**eNOS**: endothelial Nitric Oxide Synthase
**GTN**: Glyceryl trinitrate
**HEN**: Hepes, EDTA, Neocuproine
**HPDP-biotin**: N-[6-(biotinamido)hexyl]-3′-(2′-pyridyldithio)propionamide
**IFN-γ:** Interferon gamma
**iNOS**: inducible Nitric Oxide Synthase
**MMTS**: Methyl methanethiosulfonate
**MTSEA**: 2-((6-((biotinoyl)amino)-hexanoyl)amino) ethylmethanethiosulfonate
**nNOS**: neural Nitric Oxide Synthase
**NO**: Nitric Oxide
**NOS**: NO Synthase
**PBS**: Phosphate-Buffered Saline
**PLA**: Proximity Ligation Assay
**SDS**: Sodium Dodecyl Sulfate
**SDS-PAGE**: Sodium Dodecyl Sulfate-PolyAcrylamide Gel Electrophoresis
**SNO**: S-nitrosylation
**SNOC**: S-nitrosocysteine
**SNO-RAC**: S-nitrosothiol Resin Assisted Capture
**TMT**: Tandem Mass Tag
**TNFR1**: Tumor Necrosis Factor Receptor 1
**w/o**: without

## 1 Introduction

Nitric oxide (NO) is a small gaseous free radical in biological systems. Due to its lipophilic properties, NO can easily cross membranes (plasma membrane and organelle membranes) and reacts with a multitude of proteins as both an intracellular and extracellular mediator [1]. NO plays a major role in signaling pathways that regulate a diverse array of physiological processes [[2], [3], [4]]. NO is involved in the control of the cardiovascular system by promoting vasorelaxation of smooth muscle cells and vascular permeability. In the peripheral nervous system, NO acts as a neurotransmitter. In the immune system, NO participates in non-specific host defense via by macrophages, inhibits leukocyte adhesion to the endothelium, prevents monocyte chemotaxis and platelet adhesion and aggregation. NO also plays a role in regulating cell proliferation and apoptosis [2]. Furthermore, NO is involved in various pathophysiological conditions such as arthritis, atherosclerosis, diabetes, many degenerative neuronal diseases, stroke, myocardial infarction, and cancer [[5], [6], [7]].

In mammals, in normal physiology, NO is produced endogenously by a family of enzymes called NO synthases (NOS). NOS exist as three isoforms: neural NOS (nNOS or NOS1), endothelial NOS (eNOS or NOS3) and inducible NOS (iNOS or NOS2) [8,9]. Both nNOS and eNOS are constitutively expressed and their activity depends on calcium (Ca_2_^+^) concentration. These NOS enzymes release low levels of NO (nM), mainly in central and peripheral neurons (nNOS) and endothelial cells (eNOS). In contrast, iNOS is a transcriptionally regulated enzyme (in response to cytokines such as IL-1β or lipopolysaccharide along with interferon gamma (IFN-γ)) that exerts its activity in a calcium-independent manner. In contrast, iNOS can produce large quantities of NO (μM) over a long period of time. To increase the level of NO within the body, NO can be delivered exogenously through various families of NO-precursors and NO-direct-releasing drugs [10]. Among them, the most widely used NO donors in pre-clinical and clinical studies are organic nitrates and S-nitrosothiols [10]. Glyceryl trinitrate (GTN), an organic nitrate, is metabolized by enzymatic and non-enzymatic pathways to release NO [11]. In contrast, S-nitrosocysteine (SNOC), an S-nitrosothiol, is highly unstable and spontaneously releases NO in aqueous solution. However, the ability of S-nitrosothiols to release NO varies and is influenced by the local reducing environment as well as the activity of the denitrosylation pathways.

NO exerts its effects mainly via post-translational modifications (PTM) in proteins such as metal-nitrosylation, nitration and S-nitrosylation. S-nitrosylation is the major PTM induced by NO. It involves the covalent but reversible attachment of a NO moiety to the thiol group (-SH) of a cysteine within proteins, resulting in the formation of S-nitrosothiol (-S-NO) [12]. S-nitrosylation can modify protein conformation, localisation, function and stability, as well as protein-protein interactions [13].

**Fig. 1:**
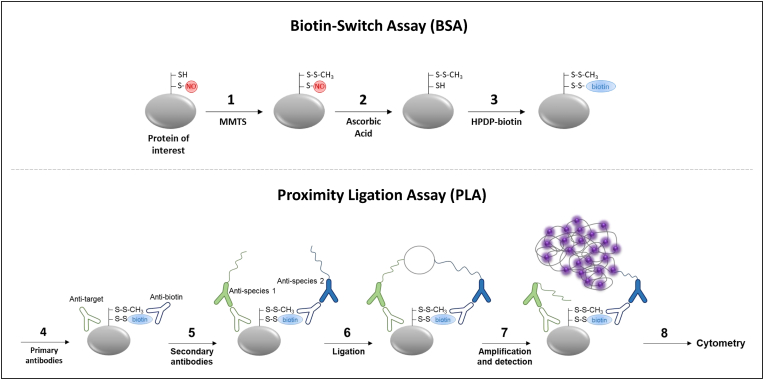
Schematic representation of the “SNO-Biotin-PLA” technique coupling Biotin-Switch Technique with Proximity Ligation Assay for the detection of S-nitrosylated proteins. The first three steps are based on the BST method, while the last five steps use the PLA technique. 1. Free thiol groups (which have not been S-nitrosylated) are blocked by methyl methanethiosulfonate (MMTS). 2. S-nitrosothiol (-S-NO) groups are reduced to free thiol groups by ascorbic acid. 3. Thiol groups (initially corresponding to S-nitrosylated sites) are biotinylated by HPDP-biotin. 4. Protein of interest and biotin are recognized by two specific primary antibodies generated from two distinct host species (e.g. species 1 anti-biotin and species 2 anti-target). 5. PLA probes (secondary antibodies coupled with oligonucleotides PLUS or MINUS) recognize primary antibodies from two different species (anti-species 1 and anti-species 2). 6. Only in close proximity, PLA probes are hybridized by connector oligos and are ligated by ligase to form a closed circular DNA template. 7. The DNA template is amplified by DNA polymerase and the resulting amplicons are detected using labelled oligonucleotides that hybridize to the repeated sequences. 8. The signal is quantified by flow cytometry analysis.

The reversible nature of the -S-NO bond (light and redox sensitive) makes S-nitrosylation a difficult PTM to analyse. Biotin-Switch Technique (BST) was developed to overcome this limitation to study protein S-nitrosylation, and it remains today the reference technique. Briefly, S-nitrosylated proteins are specifically labelled with a biotin conjugated fragment. In the first step, the free thiol groups (-SH) are blocked using a thiol-specific methylthiolating agent, methyl methanethiosulfonate (MMTS), in the presence of sodium dodecyl sulfate (SDS), which acts by denaturing proteins, exposing embedded cysteine residues. In the second step, nitrosothiol (-S-NO) bonds are selectively broken and reduced to free thiols (-SH) by the action of ascorbic acid. In the third step, the newly formed thiols are labelled with biotinylation reagent, HPDP-biotin (N-[6-(biotinamido)hexyl]-3′-(2′-pyridyldithio)propionamide) [14]. The HPDP-conjugated fragment reacts with the –SH group of cysteines to form a stable covalent bond (Fig. 1 – upper part). In the last step, biotinylated proteins are purified using the biotin/streptavidin- or neutravidin-conjugated beads affinity techniques and then analysed by Western blotting (using a primary antibody against the protein of interest) or by mass spectrometry-based techniques for identifying S-nitrosylated proteins and/or -S-NO sites.

Although BST is the gold standard technique for assessing protein S-nitrosylation, this method presents several experimental limitations. The assay can be performed on a wide range of protein amount (0.25 to 5 mg of total protein required per experimental condition) [15,16]. For example, to use 5 mg of protein, a substantial number of cells (50 x 10^6^) is necessary per sample. A large number of cells makes it challenging to process multiple samples in parallel. This is particularly limiting in cases where valuable pharmacological molecules to be tested are limited. Above all, this technique is performed on protein lysates and not *in cellulo* under physiological conditions.

Following the development of the BST, other strategies closely related to the BST have emerged to facilitate the detection of protein S-nitrosylation. Fluorescence-based assays and His-tag-based assays, have been developed to label S-nitrosylated thiol groups with fluorescent dyes or a His-tag instead of the HPDP-biotin tag. The fluorescence-switch technique eliminates the enrichment step and enables direct detection, identification and quantification of S-nitrosylated proteins using two-dimensional gel electrophoresis followed or not by mass spectrometry analysis [[17], [18], [19]]. His-tag switch offers another alternative method: the poly-histidine tag ((His)_6_ sequence) allows purification of tagged proteins using affinity chromatography columns (nickel columns) [20]. Moreover, the BST technique can be adapted with some changes to detect global S-nitrosylated proteins *in situ* within cells or tissue sections and visualised by using fluorescence microscopy. To achieve this, for example, the dye 2-((6-((biotinoyl)amino)-hexanoyl)amino) ethylmethanethiosulfonate (MTSEA)-texas red (used in replacement of HPDP-biotin) [21] or streptavidin-FITC can be used to detect all biotinylated proteins [22].

On the other hand, efforts have been made to globally profile S-nitrosylated proteins by mass spectrometry. Several SNO-proteome strategies have been developed to selectively capture and identify S-nitrosylated proteins from cell lysates. The use of a thiol-reactive resin with the so-called S-nitrosothiol Resin Assisted Capture (SNO-RAC) method is more efficient at capturing high molecular weight proteins than the BST or iodoacetyl-Tandem Mass Tag (TMT)-based immunoprecipitation methods [23]. Basically, the SNO-RAC method captures thiols (ascorbic acid mediating the conversion of S-nitrosothiols to newly formed free thiols) followed by either on-resin protein digestion and mass spectrometry analysis or protein separation by Sodium Dodecyl Sulfate-PolyAcrylamide Gel Electrophoresis (SDS-PAGE) and visualization by Western blotting or silver staining.

Although various techniques are available for the detection and identification of S-nitrosylated proteins, an experimental technique, complementary to the others, for the selective detection of the PTM S-nitrosylation *in situ*, at endogenous protein levels, under physiological conditions is missing. Hence, a new BST-related technique is presented here to detect *in situ* the S-nitrosylation of a protein of interest*.* This new method, which partially combines BST with the *Duolink®* Proximity Ligation Assay (PLA) is called “SNO-Biotin-PLA”.

## 2 Material and methods

### 2.1 Protocol for intracellular analysis of S-nitrosylation using a biotin switch technique-proximity ligation assay-combined method

Variations to the protocol were made when indicated for the analysis of transmembrane protein S-nitrosylation on the extracellular side.

1. **Treatment: seed and treat 2 x 10^6^** **cells with or without a NO donor protected from light as indicated below.**
  - *Human colorectal cancer cell lines: SW620 and SW480 were obtained from ATCC. All cell lines were cultured in DMEM supplemented with 10% of fetal bovine serum (FBS) (37 °C, 5% CO*_*2*_*),and were tested and certified as mycoplasma-free before use.*
  - *SNOC treatment:* 1mM *for* 15min *(prepared as previously described* [24].
  - *Nitronal® treatment:* 500μM *for 6-48h (Pohl-Boskamp, Hohen-lockstedt, Germany,* 1 mg/mL*), in which the pharmacologically active ingredient is GTN.*

The samples are protected from light from steps 1 to 6.

All required dilutions are performed in high purity water.

2. Collect and centrifuge the samples at 400 *g* for 5 min at 4 °C. Wash the samples two times with 1 mL of 1X phosphate-buffered saline (PBS), centrifuge at 400 *g* for 5 min at 4 °C, and remove residual 1X PBS from cells.
3. Fixation and permeabilization: resuspend cells in 100 μL of BD Cytofix/Cytoperm™ solution (BD Biosciences, product No. 554714) to fix and permeabilize samples and incubate for 20 min at 4 °C.

Note: for analysis of transmembrane protein S-nitrosylation on extracellular domains (i) skip this step and proceed to step 4; and (ii) perform all washes in steps 4 to 15 with 1X PBS containing 0.4 mM EDTA and 0.04 mM Neocuproine.

4. Following fixation and permeabilization, wash the samples two times with 0.5 mL of BST wash buffer, centrifuge at 400 *g* for 5 min at 4 °C, and remove residual wash buffer from cells.

Note: BST wash buffer consists of 1X BD Perm/Wash™ buffer (BD Biosciences, product No. 554723) containing 0.4 mM EDTA and 0.04 mM Neocuproine.

5. Blocking free thiols: resuspend cells in 1 mL of blocking buffer (prepared immediately prior to use) and incubate by mixing samples for 20 min at 50 °C to block free thiols.

Note: it is recommended to use an Eppendorf ThermoMixer® for heating and mixing samples. Otherwise, the tubes must be gently inverted at least 4 times to ensure proper mixing.

HEN buffer (250 mM HEPES pH 7.7, 1 mM EDTA, 0.1 mM Neocuproine)

Blocking buffer (20 mM MMTS (Sigma-Aldrich, product No. 64306) in HEN buffer).

6. Following blocking free thiols, wash the samples two times with 0.5 mL of BST wash buffer, centrifuge at 400 *g* for 5 min at 4 °C, and remove residual wash buffer from cells.

Note: BST wash buffer consists of 1X BD Perm/Wash™ buffer (BD Biosciences, product No. 554723) containing 0.4 mM EDTA and 0.04 mM Neocuproine.

7. Biotinylation: resuspend cells in 1 mL of HEN buffer containing 1 mM ascorbate and 0.4 mM of HPDP-biotin (EZ-Link® HPDP-biotin, ThermoFisher Scientific, product No. 21341) and incubate for 1h at room temperature using an orbital shaker to mix the samples.

Note: HPDP-biotin can be omitted from some samples as a control.

8. Following biotinylation, wash the samples two times with 0.5 mL of 1X PBS, centrifuge at 400 *g* for 5 min at 4 °C, and remove residual wash buffer from cells.

The following steps describe the Duolink® PLA flow cytometry protocol according to the manufacturer instructions (Merck, Sigma-Aldrich).

9. Blocking: resuspend cells in 30 μL of blocking solution (≈1 drop of the Duolink® Blocking Solution) and incubate for 1h at 37 °C. Aliquot 20 μL of each sample cells in final volume 100 μL 1X PBS per well of a V-bottom plate. Centrifuge the plate at 400 *g* for 5 min at room temperature and remove the Duolink® Blocking Solution from the cells.

Note: the Duolink® blocking solution is provided with the Duolink® PLA probes.

10. Primary antibodies incubation: add 100 μL of the primary antibodies solution to each sample and mix well. Incubate the plate overnight at 4 °C.

Note 1: dilute primary antibodies to suitable concentration in the Duolink® Antibody Diluent provided with the Duolink® PLA probes.

Note 2: one antibody is directed against the protein of interest and the second one is directed against biotin. It is required that the two primary antibodies come from different host species.

11. Duolink® PLA Probes incubation: centrifuge the plate at 400 *g* for 5 min at room temperature and remove the solution from the cells. Wash the cells twice with 200 μL of Duolink® Wash Buffer per well, centrifuge at 400 *g* for 5 min at room temperature, and remove the solution from the cells. Add 100 μL of the PLA probe solution and mix well. Incubate in a 37 °C incubator for 1h.

Note 1: to prepare the PLA probe solution, dilute the PLUS and MINUS PLA probes 1:5 in the Duolink® Antibody Diluent. For a 100 μL reaction, take 20 μL of PLA probe MINUS stock, 20 μL of PLA probe PLUS stock and 60 μL of Duolink® Antibody Diluent. Make sufficient solution for all samples.

Note 2: Duolink® Wash Buffer should be made prior to beginning the assay by dissolving the contents of one pouch in high purity water to a final volume of 1000 mL. Wash buffer may be stored at room temperature for short term storage (less than two weeks) or at 4 °C for long term storage.

12. Ligation: centrifuge the plate at 400 *g* for 5 min at room temperature and remove the solution from the cells. Wash the cells twice with 200 μL of Duolink® Wash Buffer per well, centrifuge at 400 *g* for 5 min at room temperature, and remove the solution from the cells. Add 100 μL of ligation solution for each cell sample and mix well. Incubate in a 37 °C incubator for 30 min.

Note 1: vortex the 5X Duolink® Ligation buffer. Dilute the 5X Ligation buffer 1:5 in high purity water and mix. For a 100 μL reaction, add 20 μL of the 5X Ligation buffer to 77.5 μL of high purity water. Make sufficient solution for all samples.

Note 2: During the wash, retrieve the Ligase from the freezer using a freezer block (−20 °C). Add Ligase to the 1X Ligation buffer at a 1:40 dilution and mix. For 100 μL ligation solution, add 2.5 μL of Ligase to 97.5 μL of the 1X ligation buffer.

13. Amplification: centrifuge the plate at 400 *g* for 5 min at room temperature and remove the solution from the cells. Wash the cells twice with 200 μL of Duolink® Wash Buffer per well, centrifuge at 400 *g* for 5 min at room temperature, and remove the solution from the cells. Add 100 μL of amplification solution for each cell samples and mix well. Incubate in a 37 °C incubator overnight.

Note 1: wait to add the polymerase until immediately prior to addition to the sample. Vortex the 5X Duolink® Amplification buffer. Dilute the 5X Amplification buffer 1:5 in high purity water and mix. For 100 μL reaction, add 20 μL of the 5X Amplification buffer to 78.75 μL of high purity water. Make sufficient solution for all samples.

Note 2: During the wash, retrieve the Polymerase from the freezer using a freezer block (−20 °C). Add Polymerase to the 1X Amplification buffer at a 1:80 dilution and mix. For 100 μL amplification solution, add 1.25 μL of Polymerase to 98.75 μL of the 1X amplification buffer.

Note 3: amplification times can be adjusted (from 100 min to overnight) depending on protein abundance or protein interactions.

14. Detection: centrifuge the plate at 400 *g* for 5 min at room temperature and remove the solution from the cells. Wash the cells twice with 200 μL of Duolink® Wash Buffer per well, centrifuge at 400 *g* for 5 min at room temperature, and remove the solution from the cells. Add 100 μL of the Duolink® Detection Buffer and mix well. Incubate in a 37 °C incubator for 60 min.

*Note 1: the Duolink® Detection Buffer is light-sensitive. Protect from light.* Vortex the 5X Detection Buffer. Dilute 1:5 in high purity water and mix. For 100 μL reaction, add 20 μL of the 5X Detection buffer to 80 μL of high purity water. Make sufficient solution for all samples.

Note 2: detection times can be adjusted (from 10 to 60 min) depending on protein abundance or level of background.

15. Final washes: centrifuge the plate at 400 *g* for 5 min at room temperature and remove the solution from the cells. Wash the cells with 200 μL of Duolink® Wash Buffer per well, centrifuge at 400 *g* for 5 min at room temperature, and remove the solution from the cells. Resuspend the cells in 300 μL 1X PBS and transfer to a flow cytometer tube. Use a flow cytometer with appropriate filters and software for data analysis.

“SNO-Biotin-PLA” experiments were performed at the ImaFlow Facility part of the US58 BioSanD.

## 3 Results

The “SNO-Biotin-PLA” technique set up here enables the study of the S-nitrosylation of a protein of interest *in situ*. This new technical approach for the detection of S-nitrosylated proteins relies on the combination of both the BST and PLA techniques which can be completed in approximately 1 to 1.5 days. The BST consists of replacing the reversible and light-sensitive-S-NO bond with covalently bound HPDP-biotin. Usually, the PLA technique can be used to detect either protein-protein interactions or protein-post-translational modifications. Here, the PLA technique is adapted to detect a biotinylated protein of interest (*i.e.* indirect detection of an S-nitrosylated protein). The technique shown here is illustrated with the detection of S-nitrosylated protein at the plasma membrane or within the cytoplasm. Notably, through this “dual” technique, we highlight here the identification of S-nitrosylation events that occur either in the intracellular or extracellular domains of a transmembrane protein of interest.

For the detection of S-nitrosylated proteins at the plasma membrane, the methodology is adapted depending on whether the S-nitrosylation to identify occurs on cysteine residues localised within the intracellular domain (fixed and permeabilized cells) or the extracellular domain (unfixed and non-permeabilized cells). First of all, cells are pre-treated with a NO donor (SNOC or Nitronal®/GTN) to generate S-nitrosylation sites. The first three steps of the “SNO-Biotin-PLA” technique are meant to replace the -S-NO bond with HPDP-biotin (Fig. 1, steps 1 to 3). The following five steps detect biotinylation of the protein of interest using two specific primary antibodies (“anti-target” and “anti-biotin”, from two distinct host species) and two modified secondary antibodies (Duolink® PLA probes) (Fig. 1, steps 4 and 5). If in close proximity, the PLA probes are hybridized through connectors (Fig. 1, step 6). The circular-like DNA template formed is amplified and detected using labelled oligonucleotides that recognize the amplicon repeats (Fig. 1, step 7). The signal is then quantified by flow cytometry analysis (Fig. 1, step 8).

The “SNO-Biotin-PLA” is illustrated here using human colorectal cancer cell lines for the detection of S-nitrosylated proteins. The S-nitrosylation of Tumor Necrosis Factor Receptor 1 (TNFR1) was previously reported by the BST and used as a positive control [25]. SW620 cells were treated or not with a NO donor (Nitronal®) for 24 h and analysed using the “SNO-Biotin-PLA” technique as described above. All the following experiments for the analysis of the S-nitrosylation of TNFR1 were performed using primary antibodies, anti-TNFR1 (directed against the intracellular domain of TNFR1, Huabio, ref. HA500140; or directed against the extracellular domain of TNFR1, Biorbyt, ref. orb100329) and anti-biotin (Biolegend, ref. 409002); and Duolink® PLA Probes MINUS-mouse PLA probe from Merck Sigma-Aldrich (ref. DUO92004) and PLUS-rabbit PLA probe (ref. DUO92002).

**Fig. 2:**
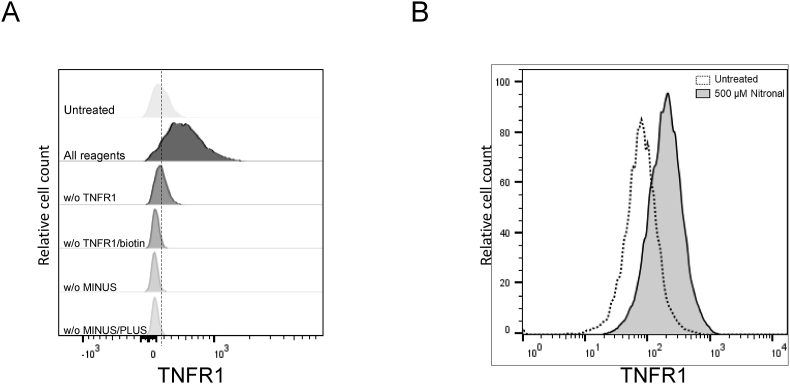
Validation of the specificity of the “SNO-Biotin-PLA” technique. A) SW620 cancer cells were either left untreated or exposed to 500 μM of Nitronal® during 24h at 37 °C. The “SNO-Biotin-PLA” technique was performed as detailed above to detect the S-nitrosylation of TNFR1 (all reagents). Negative controls conditions were performed by omitting specific reagents of the “SNO-Biotin-PLA” protocol including the omission of the TNFR1 primary antibody (w/o TNFR1), without the use of the two primary antibodies (w/o TNFR1/biotin), without the use of the MINUS-mouse PLA probe (w/o MINUS) or without the use of two PLA probes (w/o MINUS/PLUS). S-nitrosylation of intracellular domain of TNFR1 receptor was analysed with a BD® LSR II flow cytometer (BD Biosciences) using a purple laser (450 nm). One representative result from three independent experiments. B) Positive control (S-nitrosylation of intracellular domain of TNFR1 receptor) analysed with a BD LSRFortessa™ Cell Analyzer (BD Biosciences) using a purple laser (450 nm). One representative result from three independent experiments.

The specificity of the method was assessed by omitting critical reagents required within the “SNO-Biotin-PLA” technique: without (w/o) primary antibodies or PLA probes. All negative controls tested showed the expected outcome, with results comparable to untreated cells (Fig. 2A). In contrast, treatment of SW620 cells with the NO donor and subsequently labelled with all reagents exhibited a positive (Fig. 2A and B). Altogether, these results validate the ability of the “SNO-Biotin-PLA” technique to specifically detect the S-nitrosylation of the TNFR1 receptor in SW620 cells.

**Fig. 3:**
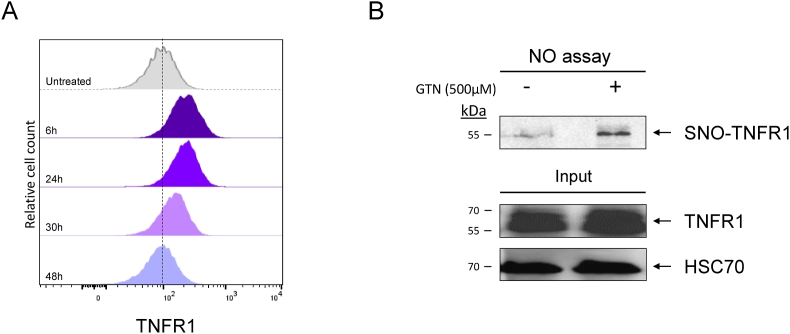
Kinetics of TNFR1 receptor S-nitrosylation in SW620 cancer cells. A) SW620 cancer cells were treated with 500 μM of Nitronal® during 6h, 24h, 30h or 48h at 37 °C or left untreated. S-nitrosylation of the intracellular domain of TNFR1 receptor was labelled by “SNO-Biotin-PLA” technique and analysed with a BD LSRFortessa™ Cell Analyzer (BD Biosciences) using a purple laser (450 nm). One representative result from three independent experiments. B) The S-nitrosylation of TNFR1 was detected in SW620 cells treated with 500 μM of GTN during 6h using the BST approach as previously described [24]. Both biotin-pull down samples and total cell lysates (input) were immunoblotted with antibodies directed against TNFR1 (Cell signaling, ref. 3736, dilution 1/2500) and HSC70 (Santa Cruz Biotechnology, ref. sc-7298, dilution 1/1000) as a loading control.

S-nitrosylation is a post-translational modification that can be reversed by two possible mechanisms: *trans*-nitrosylation and denitrosylation [26,27]. For this reason, the level of intracellular S-nitrosylation is a highly variable process over time. To study the kinetics of TNFR1 receptor S-nitrosylation, SW620 cells were treated or not (untreated) with a NO donor (Nitronal®) for 6h, 24h, 30h or 48h. The “SNO-Biotin-PLA” technique was performed and the samples were subsequently analysed by flow cytometry. A maximal positive signal, corresponding to S-nitrosylation of the TNFR1 receptor, was reached at 6h and 24h after treatment with Nitronal®, followed by a decrease in the signal over time, returning to basal levels within 48h of treatment (Fig. 3A). To validate this new approach, TNFR1 S-nitrosylation was detected in parallel by BST in SW620 cells treated with an NO donor for 6h confirming the reliability of the method (Fig. 3B).

**Fig. 4:**
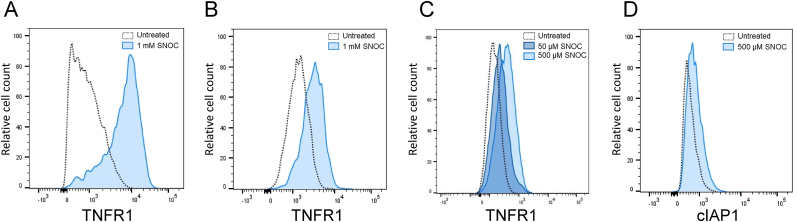
Detection of TNFR1 receptor and cytoplasmic cIAP1 S-nitrosylation. SW620 cancer cells were treated with SNOC at the indicated concentrations for 15 min at 37 °C. TNFR1 S-nitrosylation was analysed by “SNO-Biotin-PLA” technique and flow cytometry analysis using a BD LSRFortessa™ Cell Analyzer (BD Biosciences) with the yellow-green laser (586 nm) in A and purple laser (450 nm) in B and C. A) S-nitrosylation of extracellular part of the TNFR1 in unfixed, non-permeabilized SW620 cells with SNOC 1 mM. B) S-nitrosylation of intracellular part of the TNFR1 receptor in fixed and permeabilized cells with SNOC 1 mM. C) S-nitrosylation of the intracellular part of the TNFR1 receptor in fixed and permeabilized cells treated with SNOC 50 μM and 500 μM. One representative result from three independent experiments. D) SW480 cancer cells were treated with 500 μM SNOC for 15 min at 37 °C. S-nitrosylation of cIAP1 was analysed by “SNO-Biotin-PLA” technique using anti-cIAP1 (Biorbyt, ref. orb402256, dilution 1/250) and anti-biotin (Huabio, ref. HA500140, dilution 1/500) followed by cytometry analysis. Cytoplasmic cIAP1 S-nitrosylation was analysed using a BD LSRFortessa™ Cell Analyzer (BD Biosciences) with the purple laser (450 nm). One representative result from three independent experiments.

To further develop the “SNO-Biotin-PLA” technique, experiments were performed using a different NO donor. The NO donor SNOC (an S-nitrosothiol that spontaneously releases a large quantity of NO in an aqueous solution) was used instead of Nitronal® (which is metabolized to release NO). SW620 cells, either left untreated or treated at the indicated concentrations with SNOC for 15 min, were analysed using the “SNO-Biotin-PLA” technique to detect the S-nitrosylation of the TNFR1 extracellular domain (Fig. 4A) and the TNFR1 intracellular domain (Fig. 4B and C). Interestingly, the signal intensity appears higher when studying S-nitrosylation of the extracellular domain of the TNFR1 receptor (Fig. 4A) compared to its intracellular domain (Fig. 4B). These results may be related to the fact that the extracellular domain of the TNFR1 receptor is rich in cysteines (24 cysteines versus 5 in the intracellular domain), which are critical target sites for NO. In addition, the level of S-nitrosylation of TNFR1 appears to increase in a dose-dependent manner with higher levels observed at 1 mM (Fig. 4B) compared to lower concentrations (50 μM-500μM) of SNOC (Fig. 4C).

Finally, the feasibility of the technique was also confirmed in another cell line, with a different target protein. In our previous report, we demonstrated the S-nitrosylation of cIAP1 upon an NO donor treatment, as demonstrated using the reference BST [24]. Here, we confirmed the S-nitrosylation of cIAP1, as a cytoplasmic protein, in the human colon cancer cell line SW480 using the “SNO-Biotin-PLA” technique (Fig. 4D).

Taken together, these results confirm that the “SNO-Biotin-PLA” technique enables selective *in situ* detection of protein S-nitrosylation using various NO donors.

## 4 Discussion

Nitric oxide (NO) is a crucial signaling molecule in many physiological and pathological processes. The development of new techniques to study NO-mediated PTMs could have significant implications for the development of innovative therapies for clinical applications. Therefore, new approaches are needed to gain deeper insights into the complex role of NO in cellular signaling and to support the development of novel therapeutic strategies targeting NO pathways.

The development of the PLA technique has significantly advanced the study of protein-protein interactions and PTMs over traditional methods (such as immunofluorescence co-localisation or immunoprecipitation). Identifying the PTMs of individual proteins is of major interest for understanding their role in cellular processes and the regulation of their activity. The PLA approach enables high sensitivity and specificity for *in situ* protein detection, making it a widely used method for this purpose.

Nevertheless, like any experimental technique, PLA exhibits inherent limitations. As an antibody-based technique, the reliability of the results depends on the quality and specificity of the antibodies. Therefore, the use of well-characterized antibodies is of utmost importance. Antibody affinity and accessibility to the relevant epitopes can ideally be validated through knock-out and flow cytometry analyses.

PLA is based on the generation of a signal through a proximity-dependent enzymatic ligation reaction. The resolution of the technique is limited within a detection radius of less than 40 nm, meaning that the signal reflects the close proximity of the PLUS and MINUS PLA probes used. However, the PLA can be prone to false positive results under certain experimental conditions. This limitation has been particularly reported when transfected overexpression systems are used to study protein-protein interactions [28]. This may be associated with irrelevant biological signals resulting from protein being overexpressed at very high levels within confined subcellular compartments.

Herein, PLA is designed to detect intrinsic PTMs inherent to a protein of interest at endogenous levels. The effective proximity between two epitopes on the same protein is theoretically much less than 40 nm. Thus, any false negative signal should not be attributed to the distance between two epitopes within a protein, but rather to other parameters such as nonspecific binding of primary antibodies, inefficient ligation or amplification. On the contrary, a putative interaction between a protein of interest with another S-nitrosylated protein (thus biotinylated) may potentially generate a false positive signal. However, given that PLA can theoretically detect epitopes separated by virtually 0 nm (as long as the two primary antibodies can access and bind their respective epitopes on the same protein) and considering the molecular characteristics of the PLA probes, the highest degree of proximity is likely favored. Consistently, findings can be validated using an alternative approach or by generating functional mutants that abolish NO-mediated PTMs.

Importantly, the present technique offers significant advantages over existing methods for the study of S-nitrosylation. It enables *in situ* analysis of S-nitrosylation, with selective *in situ* protein detection using a small number of cells, facilitating comparative analyses between different samples. This approach preserves the biological context, the spatial organization and allows the detection of transient events, providing a more accurate analysis of S-nitrosylation dynamics. Another advantage is the possibility of specifically studying the S-nitrosylation of a particular protein domain using antibodies directed against a specific relevant epitope. Importantly, this newly developed technique, derived from the BST method, involves the same light-sensitive steps, however, the duration of light protection throughout the protocol is shorter. Finally, it is a more time-efficient technique than BST as it relies on a straightforward flow cytometry analysis rather than a two-day Western Blot analysis.

Therefore, the “SNO-Biotin-PLA” represents a powerful technique for the detection of S-nitrosylated proteins. This novel approach could considerably enhance the understanding of the role of S-nitrosylation in various physiopathological processes such as inflammatory diseases, metabolic disorders, cardiovascular diseases and cancer in both basic and translational research. Given the essential role of PTMs in fundamental cellular processes as well as in pathogenesis, this novel technique may have potential applications in clinical diagnostics and therapeutics.

## CRediT authorship contribution statement

**Mélina Meunier:** Formal analysis, Investigation, Writing – review & editing. **Emma Levieux:** Formal analysis, Investigation. **Stéphanie Plenchette:** Conceptualization, Formal analysis, Methodology, Supervision, Writing – original draft, Writing – review & editing.

## Declaration of competing interest

The authors declare the following financial interests/personal relationships which may be considered as potential competing interests: Melina Meunier reports financial support was provided by Foundation for Medical Research. Stephanie Plenchette reports financial support was provided by National Institute for Cancer and SATT SAYENS. Stephanie Plenchette has patent pending to EP24307118. If there are other authors, they declare that they have no known competing financial interests or personal relationships that could have appeared to influence the work reported in this paper.

## Data Availability

Data will be made available on request.

## References

[1] Möller M.N., Denicola A. (Nov. 2018). Diffusion of nitric oxide and oxygen in lipoproteins and membranes studied by pyrene fluorescence quenching. Free Radic. Biol. Med..

[2] Holotiuk V.V., Kryzhanivska A.Y., Churpiy I.K., Tataryn B.B., Ivasiutyn D.Y. (Sep. 2019). Role of nitric oxide in pathogenesis of tumor growth and its possible application in cancer treatment. Exp. Oncol..

[3] Blaise G.A., Gauvin D., Gangal M., Authier S. (Mar. 2005). Nitric oxide, cell signaling and cell death. Toxicology.

[4] Moncada S., Palmer R.M., Higgs E.A. (Jun. 1991). Nitric oxide: physiology, pathophysiology, and pharmacology. Pharmacol. Rev..

[5] Daiber A. (Jan. 2019). New therapeutic implications of endothelial nitric oxide synthase (eNOS) function/dysfunction in cardiovascular disease. Int. J. Mol. Sci..

[6] Maher A., Abdel Rahman M.F., Gad M.Z. (2017). The role of nitric oxide from neurological disease to cancer. Adv. Exp. Med. Biol..

[7] Keshet R., Erez A. (Aug. 2018). Arginine and the metabolic regulation of nitric oxide synthesis in cancer. Dis. Model. Mech..

[8] Marletta M.A. (Sep. 1994). Nitric oxide synthase: aspects concerning structure and catalysis. Cell.

[9] Dong J., Li D., Kang L., Luo C., Wang J. (Jun. 2023). Insights into human eNOS, nNOS and iNOS structures and medicinal indications from statistical analyses of their interactions with bound compounds. Biophys. Rep..

[10] Alimoradi H., Greish K., Gamble A.B., Giles G.I. (Dec. 2019). Controlled delivery of nitric oxide for cancer therapy. Pharm. Nanotechnol..

[11] Meunier M., Yammine A., Bettaieb A., Plenchette S. (May 2023). Nitroglycerin: a comprehensive review in cancer therapy. Cell Death Dis..

[12] Stamler J.S. (Sep. 1994). Redox signaling: nitrosylation and related target interactions of nitric oxide. Cell.

[13] Furuta S. (Nov. 2017). Basal S-Nitrosylation is the guardian of tissue homeostasis. Trends Cancer.

[14] Jaffrey S.R., Snyder S.H. (Jun. 2001). The biotin switch method for the detection of S-Nitrosylated proteins. Sci. STKE.

[15] Kelleher Z.T., Matsumoto A., Stamler J.S., Marshall H.E. (Oct. 2007). NOS2 regulation of NF-kappaB by S-nitrosylation of p65. J. Biol. Chem..

[16] Forrester M.T., Foster M.W., Benhar M., Stamler J.S. (Jan. 2009). Detection of protein S-nitrosylation with the biotin-switch technique. Free Radic. Biol. Med..

[17] Tello D., Tarín C., Ahicart P., Bretón-Romero R., Lamas S., Martínez-Ruiz A. (2009). A ‘fluorescence switch’ technique increases the sensitivity of proteomic detection and identification of S-nitrosylated proteins. Proteomics.

[18] Kettenhofen N.J., Wang X., Gladwin M.T., Hogg N., Cadenas E., Packer L. (2008).

[19] Santhanam L. (Nov. 2008). Selective fluorescent labeling of S-nitrosothiols (S-FLOS): a novel method for studying S-nitrosation. Nitric Oxide.

[20] Camerini S., Polci M.L., Restuccia U., Usuelli V., Malgaroli A., Bachi A. (Aug. 2007). A novel approach to identify proteins modified by nitric oxide: the HIS-TAG switch method. J. Proteome Res..

[21] Yang Y., Loscalzo J. (Jan. 2005). S-nitrosoprotein formation and localization in endothelial cells. Proc. Natl. Acad. Sci. U. S. A.

[22] Ckless K., Reynaert N.L., Taatjes D.J., Lounsbury K.M., van der Vliet A., Janssen-Heininger Y. (Nov. 2004). In situ detection and visualization of S-nitrosylated proteins following chemical derivatization: identification of Ran GTPase as a target for S-nitrosylation. Nitric Oxide.

[23] Forrester M.T., Thompson J.W., Foster M.W., Nogueira L., Moseley M.A., Stamler J.S. (Jun. 2009). Proteomic analysis of S-nitrosylation and denitrosylation by resin-assisted capture. Nat. Biotechnol..

[24] Romagny S. (Apr. 2018). S-Nitrosylation of cIAP1 switches cancer cell fate from TNFα/TNFR1-Mediated cell survival to cell death. Cancer Res..

[25] Rodríguez-Hernández A. (Dec. 2015). Regulation of cell death receptor S-nitrosylation and apoptotic signaling by Sorafenib in hepatoblastoma cells. Redox Biol..

[26] Hess D.T., Stamler J.S. (Feb. 2012). Regulation by S-nitrosylation of protein post-translational modification. J. Biol. Chem..

[27] Stomberski C.T., Hess D.T., Stamler J.S. (Apr. 2019). Protein S-Nitrosylation: determinants of specificity and enzymatic regulation of S-Nitrosothiol-Based signaling. Antioxid. Redox Signaling.

[28] Tower Z., Chang H. (Sep. 02, 2024). bioRxiv: the Preprint Server for Biology: 2024.09.01.610697.

